# The endoplasmic reticulum remains functionally connected by vesicular transport after its fragmentation in cells expressing Z-α_1_-antitrypsin

**DOI:** 10.1096/fj.201600430R

**Published:** 2016-09-06

**Authors:** Jennifer A. Dickens, Adriana Ordóñez, Joseph E. Chambers, Alison J. Beckett, Vruti Patel, Elke Malzer, Caia S. Dominicus, Jayson Bradley, Andrew A. Peden, Ian A. Prior, David A. Lomas, Stefan J. Marciniak

**Affiliations:** * Cambridge Institute for Medical Research, Cambridge, United Kingdom;; † Department of Medicine, University of Cambridge, Cambridge, United Kingdom;; ‡ Department of Biomedical Science, University of Sheffield, Sheffield, United Kingdom;; § Institute of Translational Medicine, University of Liverpool, Liverpool, United Kingdom; and; ¶ UCL Respiratory, University College London, London, United Kingdom

**Keywords:** ER stress, homotypic fusion, serpin, SNARE

## Abstract

α_1_-Antitrypsin is a serine protease inhibitor produced in the liver that is responsible for the regulation of pulmonary inflammation. The commonest pathogenic gene mutation yields Z-α_1_-antitrypsin, which has a propensity to self-associate forming polymers that become trapped in inclusions of endoplasmic reticulum (ER). It is unclear whether these inclusions are connected to the main ER network in Z-α_1_-antitrypsin-expressing cells. Using live cell imaging, we found that despite inclusions containing an immobile matrix of polymeric α_1_-antitrypsin, small ER resident proteins can diffuse freely within them. Inclusions have many features to suggest they represent fragmented ER, and some are physically separated from the tubular ER network, yet we observed cargo to be transported between them in a cytosol-dependent fashion that is sensitive to *N*-ethylmaleimide and dependent on Sar1 and sec22B. We conclude that protein recycling occurs between ER inclusions despite their physical separation.—Dickens, J. A., Ordóñez, A., Chambers, J. E., Beckett, A. J., Patel, V., Malzer, E., Dominicus, C. S., Bradley, J., Peden, A. A., Prior, I. A., Lomas, D. A., Marciniak, S. J. The endoplasmic reticulum remains functionally connected by vesicular transport after its fragmentation in cells expressing Z-α_1_-antitrypsin.

α_1_-Antitrypsin is a serine protease inhibitor responsible for the regulation of pulmonary inflammation (1). It is secreted by hepatocytes into the plasma at high concentrations of 1 to 2 g/L, but deficiency can result from one of many mutations of the *SERPINA1* gene (2). The most common mutation in Europeans is E342K, so-called Z-α_1_-antitrypsin, which causes a subtle structural change predisposing the protein to self-associate into ordered polymers that become trapped within the synthesizing cell (3). Surprisingly, in only a minority of patients do the resulting inclusions in hepatocytes cause toxic gain of function resulting in clinically significant liver disease (4), whereas plasma deficiency and early-onset pulmonary emphysema are typical, resulting from unchecked activity of neutrophil elastase (5).

The inclusion bodies of polymerized α_1_-antitrypsin contain the endoplasmic reticulum (ER)-resident chaperones BiP and PDI, and are frequently decorated with ribosomes (6, 7). However, these inclusions appear to differ from healthy ER in other respects; for example, they have been reported to lack the chaperone calnexin (CNX) and have wide lumens of >500 nm compared to <100 nm for normal ER (7, 8). This suggests that inclusions of polymerized α_1_-antitrypsin represent aberrant ER. Indeed, it has been postulated that inclusion bodies represent ER that has been walled off to protect the main network from the polymeric α_1_-antitrypsin (7). Despite this, there is little evidence for ER stress during the accumulation of polymerized α_1_-antitrypsin or for activation of the unfolded protein response (8–10). Instead, the distension of the ER by polymerized α_1_-antitrypsin and other serine protease inhibitors (serpins) activates an ER overload response mediated by NF-κB (11). We and others have reported that polymerization of α_1_-antitrypsin within the ER leads to an exaggerated unfolded protein response if ER stress is caused by other means (8, 12). We showed that this correlates with reduced mobility of small ER marker proteins in cells containing inclusions (8). Moreover, it has been suggested that if polymers of α_1_-antitrypsin cannot be segregated into inclusions, this leads to ER stress (7). Whether inclusion bodies can communicate with one another or with the remaining ER network remains unknown. Subcellular fractionation has suggested that inclusion bodies are physically separated (7), but dynamic imaging of fluorescent marker proteins suggests that interinclusion communication might occur (8). Whether polymerized α_1_-antitrypsin can move between the ER and inclusions or between inclusions themselves remains unknown.

In this study, we sought to clarify the behavior of inclusion body contents, both soluble resident proteins and polymerized α_1_-antitrypsin. We report that the structure formed of Z-α_1_-antitrypsin within an inclusion body behaves as a matrix of poorly mobile material through which smaller proteins can readily diffuse. Remarkably, small proteins rapidly exchange between physically distinct inclusion bodies by vesicular transport that requires cytosol, is sensitive to *N*-ethylmaleimide (NEM) and dependent on Sar1 and sec22B, but is independent of atlastin-mediated homotypic fusion of the ER.

## MATERIALS AND METHODS

### Reagents

Primary antibodies used were as follows: mouse anti-total α_1_-antitrypsin monoclonal antibody (Abcam, Cambridge, MA, USA), mouse anti-polymer α_1_-antitrypsin mAb (2C1) (13), rabbit anti-myc pAb (Abcam), and rabbit anti-sec22B (gift from J. Hay, University of Montana) (14). Secondary antibodies were horseradish peroxidase–tagged anti-mouse and Texas Red–conjugated anti-rabbit and mouse antibodies (Abcam).

### Plasmid and short interfering RNA constructs

The coding sequence of wild-type M- or mutant Z-α_1_-antitrypsin was cloned 3′ to yellow fluorescent protein (YFP) in the eYFP-ER plasmid *Eco*R1 and *Sal1* sites (Clontech Laboratories, Mountain View, CA, USA). A flexible (Gly_4_Ser)_3_ linker was inserted between YFP and α_1_-antitrypsin to minimize aggregation of the fusion protein while avoiding steric effects on polymerization. HaloTag constructs were generated from this vector by inserting PCR-amplified HaloTag cDNA from pHTN HaloTag CMV-neo vector (Promega, Madison, WI, USA) between *PspXI* and *bglII* in place of YFP. pcDNA-α_1_-antitrypsin constructs were described previously (15). The Gmx33–green fluorescent protein (GFP) and mCherry-ER plasmids were gifts from M. Seaman and D. Ron, respectively (University of Cambridge, UK). Wild-type atlastin constructs were gifts from E. Reid (University of Cambridge, UK); the K80A mutant was generated by site-directed mutagenesis. The cytERM-msfGFP and BiP-mCherry constructs were gifts from E. Snapp (Albert Einstein College of Medicine, New York, USA). The GFP–reticulon 4a construct was a gift from G. Voeltz (University of Colorado, USA). The Sar1-CFP constructs were gifts from H. Maccioni (National University of Cordoba, Argentina). The CNX-mCherry construct was created by Gibson assembly with ligation of CNX, flexible linker, and mCherry sequences into an *Eco*RV-digested pcDNA3 plasmid. Sec22B and control short interfering RNAs (siRNAs) were obtained from Dharmacon (Lafayette, CO, USA) and Ambion (Austin, TX, USA), respectively.

### Cell culture and transfection

Chinese hamster ovary (CHO) cells were cultured in DMEM supplemented with 10% v/v fetal bovine serum, penicillin/streptomycin, and nonessential amino acids. Cells were transfected overnight with Lipofectamine LTX (Thermo Fisher Scientific, Waltham, MA, USA) and imaged at least 24 h after the transfection reagent was removed. For siRNA experiments, cells were transfected with 20 nM siRNA using RNAiMAX (Thermo Fisher Scientific) for 72 h. Forty-eight hours before imaging, cells were also transfected with mCherry-ER and YFP-AAT constructs.

### ELISA and Western blot analysis

Triton lysates, SDS, and nondenaturing PAGE followed by immunoblotting or ELISA were performed as detailed previously (16, 17).

### Radiolabeling and immunoprecipitation

CHO cells were labeled with [^35^S]-Met/Cys for 15 min and chased for the times indicated. α_1_-Antitrypsin from cell lysates and medium was immunoprecipitated with a polyclonal antibody, then analyzed by SDS-PAGE and autoradiography.

### Immunofluorescence

Cells were fixed with 4% v/v formaldehyde for 30 min, washed, and then permeabilized with 0.1% v/v Triton X-100 for 15 min. Plates were washed, blocked with 10% v/v fetal bovine serum for 30 min, and then incubated with primary antibody for 2 h. After a further wash, plates were incubated with a fluorochrome-conjugated secondary antibody for 1 h. After washing, cells were kept in PBS containing 3.3 µg/ml DAPI.

### Microscopy including photobleaching in live cells

CHO cells were grown in 35 mm live cell imaging dishes (MatTek, Ashland, MA, USA), transfected as required, and left in culture for 24 h after transfection before imaging. Cells were imaged in tissue culture medium supplemented with 25 mM HEPES, pH 7.0 to 7.6. Fluorescence recovery after photobleaching (FRAP) and fluorescence loss in photobleaching (FLIP) imaging was undertaken using a confocal laser scanning microscope (LSM710; Carl Zeiss GmbH, Jena, Germany) in a 37°C/5% v/v CO_2_ chamber with a ×40/1.3 NA oil objective. Zen 2010 software was used for all imaging. A small region of interest was photobleached using laser powers to achieve an approximately 50% bleach (variable by protein) using the 488 nm (GFP), 514 nm (YFP), and/or 594 nm (mCherry) lasers as necessary. Fluorescence loss or recovery was monitored with a 0.1 ms delay between images. Data from a minimum of 12 cells per condition were normalized to reference regions of interest. Confirmation of bleaching in the *z* plane was confirmed using a postbleach *z* stack. For 3-dimensional imaging, *z* stacks were taken using overlapping confocal slices, and images were reconstructed into 3-dimensional movies using Imaris software (Bitplane, Zurich, Switzerland).

### Serial block-face electron microscopy

CHO cells were transfected with YFP-Z-α_1_-antitrypsin and plated onto gridded glass-bottomed microscopy dishes. A suitable cell was identified by fluorescence microscopy. Cells were fixed and then extensively stained following OTO protocol (18). Once embedded in resin, the cell was imaged using the Gatan 3View system (Gatan, Abingdon, UK) mounted on a Quanta 250 scanning electron microscope (FEI, Cambridge, United Kingdom). A 3View stack was generated with a resolution of 18 nm in *x* and *y* and 60 nm in *z.* The stack was aligned and 3-dimensional reconstructions created using Imaris software.

### Cell fusion

After trypsinization, 5 × 10^6^ cells of each HaloTag stain or fluorescent protein were mixed, pelleted at 250*g,* and resuspended in 180 μl medium before being transferred to an electroporation cuvette (Bio-Rad, Hercules, CA, USA). Cells were repelleted, then electroporated using 220 V/900 μF in a Gene Pulser II electroporator (Bio-Rad). The cuvette was left to incubate at 37°C for 30 min; the cells were then resuspended and plated. Plates were fixed at appropriate time points using 4% v/v formaldehyde.

### Cell permeabilization

Before permeabilization, cells were washed once with ice-cold PBS, then twice with ice-cold transport buffer (TB; 40 mM HEPES pH 7.3, 110 mM potassium acetate, 5 mM sodium acetate, 2 mM magnesium acetate, 1 mM EGTA, 52 mM sucrose). Cells were permeabilized on ice for 5 min with 40 μg/ml digitonin diluted in complete transport buffer (CTB; TB with protease inhibitor cocktail [Roche, Basel, Switzerland] and 1 mM DTT), washed twice with CTB, and incubated in CTB thereafter. All FRAP experiments were performed within 30 min of permeabilization.

### Cytosol preparation

CHO cells were homogenized using an European Molecular Biology Laboratory ball bearing homogenizer, then centrifuged at 100,000 *g* for 1 h. Protease inhibitor cocktail and 1 mM DTT were added to the supernatant, which was flash-frozen in liquid nitrogen before storage at −80°C. For NEM-treated cytosol, washed cells were exposed to 20 mM NEM for 10 min at 4°C before washing thoroughly with PBS before homogenization.

### Statistical analysis

Unless otherwise specified, data are presented as means ± sem. Two-way ANOVA was used for comparison of FRAP recovery curves.

## RESULTS

### N-terminal YFP-tagged α_1_-antitrypsin behaves similarly to untagged protein

**Figure 1.**
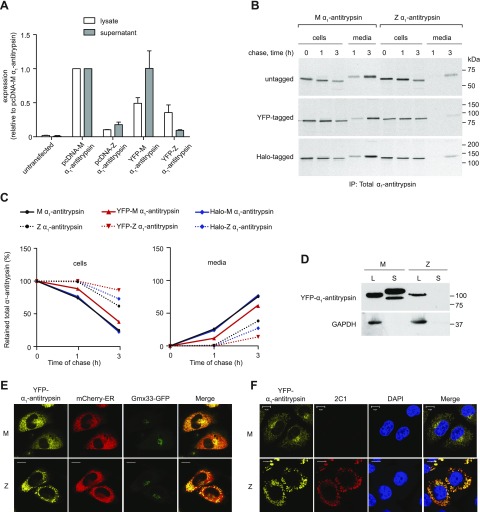
Characterization of YFP-α_1_-antitrypsin. *A*) CHO cells were transfected with untagged (pcDNA) or YFP-tagged M-α_1_-antitrypsin or Z-α_1_-antitrypsin. Whole-cell lysates and supernatants were analyzed for total α_1_-antitrypsin production by sandwich ELISA (*n* = 3). *B*) CHO cells expressing untagged α_1_-antitrypsin, YFP-α_1_-antitrypsin, or HaloTag-α_1_-antitrypsin were pulse labeled with [^35^S]-Met/Cys for 15 min and chased for 0 to 3 h. Alpha-1-antitrypsin from Triton X-100 cell lysates and medium was immunoprecipitated with polyclonal antibody; samples were then resolved by 10% SDS-PAGE and detected by autoradiography. *C*) Quantification of retained (left) and secreted (right) α_1_-antitrypsin reveal comparable profiles of untagged and tagged protein. *D*) Western blot of cell lysates (L) and medium supernatants (S) from YFP-α_1_-antitrypsin CHOs show band of expected size for YFP-α_1_-antitrypsin fusion protein. Lower-mobility mature glycoform of α_1_-antitrypsin is visible only in YFP-M-α_1_-antitrypsin-conditioned medium. *E*) CHO cells cotransfected with YFP-α_1_-antitrypsin (yellow), mCherry-ER (red), and Golgi marker Gmx33-GFP (green). YFP-M-α_1_-antitrypsin colocalized with both ER and Golgi markers, whereas YFP-Z-α_1_-antitrypsin accumulated in punctate inclusions labeled with ER marker and failed to colocalize with Golgi marker. Scale bars, 10 µm. *F*) CHO cells were transiently transfected with YFP-α_1_-antitrypsin, fixed, labeled with 2C1 antibody, and analyzed by immunofluorescence microscopy. Positive 2C1 staining indicative of pathogenic polymer formation was observed only in cells expressing YFP-Z-α_1_-antitrypsin. Scale bars, 10 µm.

To visualize α_1_-antitrypsin trafficking in live cells, we fused wild-type M- or polymerogenic Z-α_1_-antitrypsin to an ER-targeted YFP separated by a flexible (Gly_4_Ser)_3_ linker. When expressed in CHO cells, M α_1_-antitrypsin is secreted, while Z-α_1_-antitrypsin is retained within the ER as polymers (8). Cell lysates and conditioned media were examined by ELISA for total α_1_-antitrypsin (13). Similar to the untagged protein, YFP-tagged M-α_1_-antitrypsin was secreted with little intracellular retention, while YFP-Z-α_1_-antitrypsin was largely retained as intracellular inclusions (**Fig. 1*A***). The lower apparent expression of Z-α_1_-antitrypsin was due, at least in part, to its lower solubility in Triton X-100 lysis buffers, since more Z- than M-α_1_-antitrypsin could be recovered from the Triton X-100–insoluble fraction using SDS-containing buffers (data not shown). Addition of the YFP tag did not alter the time course of secretion of M- or Z-α_1_-antitrypsin, or the ability of Z-α_1_-antitrypsin to form polymers detectable by the 2C1 antibody that specifically recognizes pathogenic polymers of Z-α_1_-antitrypsin (13) (Fig. 1*B, C, F*). Cell lysates and culture media were analyzed by SDS-PAGE and immunoblotting. Proteins of the expected size were observed with only the M-α_1_-antitrypsin being detected in the medium (Fig. 1*D*). Cells were cotransfected with the tagged-α_1_-antitrypsin and with a marker of the ER (mCherry-ER) or the Golgi apparatus (Gmx33-GFP) (19) and imaged by confocal microscopy. Consistent with efficient trafficking, YFP-M-α_1_-antitrypsin colocalized with both markers (Fig. 1*E*). In contrast, YFP-Z-α_1_-antitrypsin resided in punctate structures that were positive for mCherry-ER but not Gmx33-GFP (Fig. 1*E*). From these results, we concluded that YFP-tagged α_1_-antitrypsin behaves in a fashion comparable to untagged protein.

### Inclusions contain a gel-like matrix through which small proteins can diffuse

**Figure 2.**
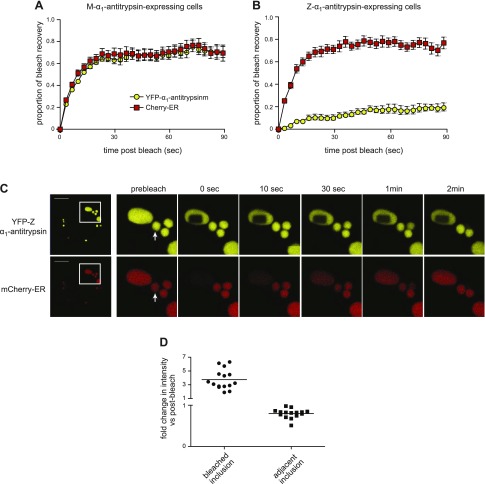
Inclusions contain gel-like material through which small proteins can move. *A*, *B*) CHO cells were cotransfected with YFP-α_1_-antitrypsin and mCherry-ER; simultaneous 2-color FRAP was then performed using region of interest 5–10% cross-sectional area of cell: YFP-M-α_1_-antitrypsin (*A*), YFP-Z-α_1_-antitrypsin (*B*). mCherry fluorescence (red squares) recovered rapidly in both YFP-M-α_1_-antitrypsin and YFP-Z-α_1_-antitrypsin-expressing cells, whereas fluorescence of YFP-Z-α_1_-antitrypsin (yellow circles) recovered more slowly than that of YFP-M-α_1_-antitrypsin. For YFP-Z-α_1_-antitrypsin-expressing cells, number of inclusions was included within bleached area. *C*) Two-color FRAP was performed with region of interest sufficiently small to lie within one large inclusion. Area of bleached YFP-Z-α_1_-antitrypsin persists more than 2 min; however, mCherry fluorescence was completely bleached within inclusion but recovered during 2 min, while adjacent inclusions partially dimmed. Scale bars, 10 µm. Adjacent inclusion used for quantitation (*D*) is indicated with arrow. *D*) Quantification of mCherry fluorescence intensity in bleached *vs.* adjacent inclusion 30 s after bleaching (relative to immediate postbleaching intensity).

We next examined the interior environment of inclusions of α_1_-antitrypsin. Cells coexpressing YFP-α_1_-antitrypsin and the small ER-resident marker protein mCherry-ER were subjected to 2-color FRAP. As expected, in M-α_1_-antitrypsin-expressing cells, we observed rapid recovery of both YFP-M-α_1_-antitrypsin and mCherry-ER consistent with free movement of these proteins within a continuous ER compartment (**Fig. 2*A***). In contrast, in Z-α_1_-antitrypsin-expressing cells, the recovery of YFP-Z-α_1_-antitrypsin was minimal (Fig. 2*B*, yellow) but unexpectedly, the mCherry-ER fluorescence recovered rapidly (Fig. 2*B*, red). Because these data suggested that small proteins might be able to diffuse within a matrix of Z-α_1_-antitrypsin, we examined protein mobility in a single inclusion (Fig. 2*C* and Supplemental Movie 1). The resulting bleached region of YFP-Z-α_1_-antitrypsin in one inclusion remained well defined at 2 min after bleaching. In marked contrast, mCherry-ER was bleached completely within the entire inclusion during delivery of 200 iterations of laser bleaching (Fig. 2*C*, bottom). Such a result would be expected for a rapidly diffusing protein and suggests that despite the relative immobility of Z-α_1_-antitrypsin, mCherry-ER can move freely within the same volume. The fluorescence signal of mCherry-ER then recovered gradually, while nearby inclusions showed a modest loss of signal. To study the movement of fluorescent protein between inclusions, we measured the relative mCherry fluorescence intensities in the bleached *vs.* the adjacent inclusion 30 s after bleaching (Fig. 2*D*). The fluorescence recovering into the bleached inclusion corresponded to fluorescence loss from the adjacent inclusion, suggesting exchange of protein between inclusions.

**Figure 3.**
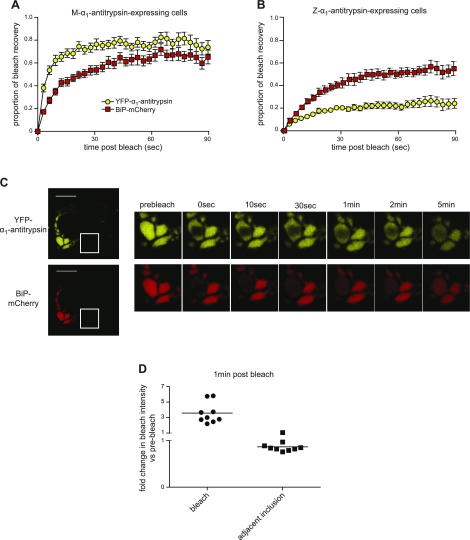
Mobility of ER chaperone BiP is retarded in Z-α_1_-antitrypsin-expressing cells. CHO cells coexpressing BiP-mCherry and either YFP-M-α_1_-antitrypsin (*A*) or YFP-Z-α_1_-antitrypsin (*B*) were subjected to 2-color FRAP. Retardation of recovery of BiP-mCherry fluorescence (red squares) was noted in YFP-Z-α_1_-antitrypsin *vs.* YFP-M-α_1_-antitrypsin-expressing cells (*P* < 0.0001). *C*) Two-color FRAP was performed with region of interest sufficiently small to lie within one large inclusion. Area of bleached YFP-Z-α_1_-antitrypsin persists for more than 2 min. BiP-mCherry fluorescence was completely bleached within inclusion but began to recover during 5 min, while adjacent inclusions partially dimmed. Scale bars, 10 µm. *D*) Quantification of BiP-mCherry fluorescence intensity in bleached *vs.* adjacent inclusion 1 min after bleaching relative to immediate postbleaching intensity.

We next examined the mobility of the abundant ER chaperone BiP by using a mCherry-tagged construct (20). As we had observed for mCherry-ER, BiP-mCherry was mobile within and between inclusions (**Fig. 3**). Its mobility was modestly reduced in cells expressing M-α_1_-antitrypsin, which might reflect its larger size and/or interaction with client proteins (Fig. 3*A*). This reduction in mobility was more marked in cells expressing Z-α_1_-antitrypsin (Fig. 3*B*). Nevertheless, BiP-mCherry remained mobile when observed within a single inclusion (Fig. 3*C*). Although the bleached region of YFP-Z-α_1_-antitrypsin remained visible for up to 5 min after bleaching, the overexpression of BiP-mCherry appeared to increase the mobility of α_1_-antitrypsin within inclusions (Fig. 3*B, C*). As we had observed for mCherry-ER, during the bleaching procedure, there was uniform loss of BiP-mCherry fluorescence. Once again, dimming of adjacent inclusions followed this (Fig. 3*C, D*).

### Inclusions are physically disconnected

**Figure 4.**
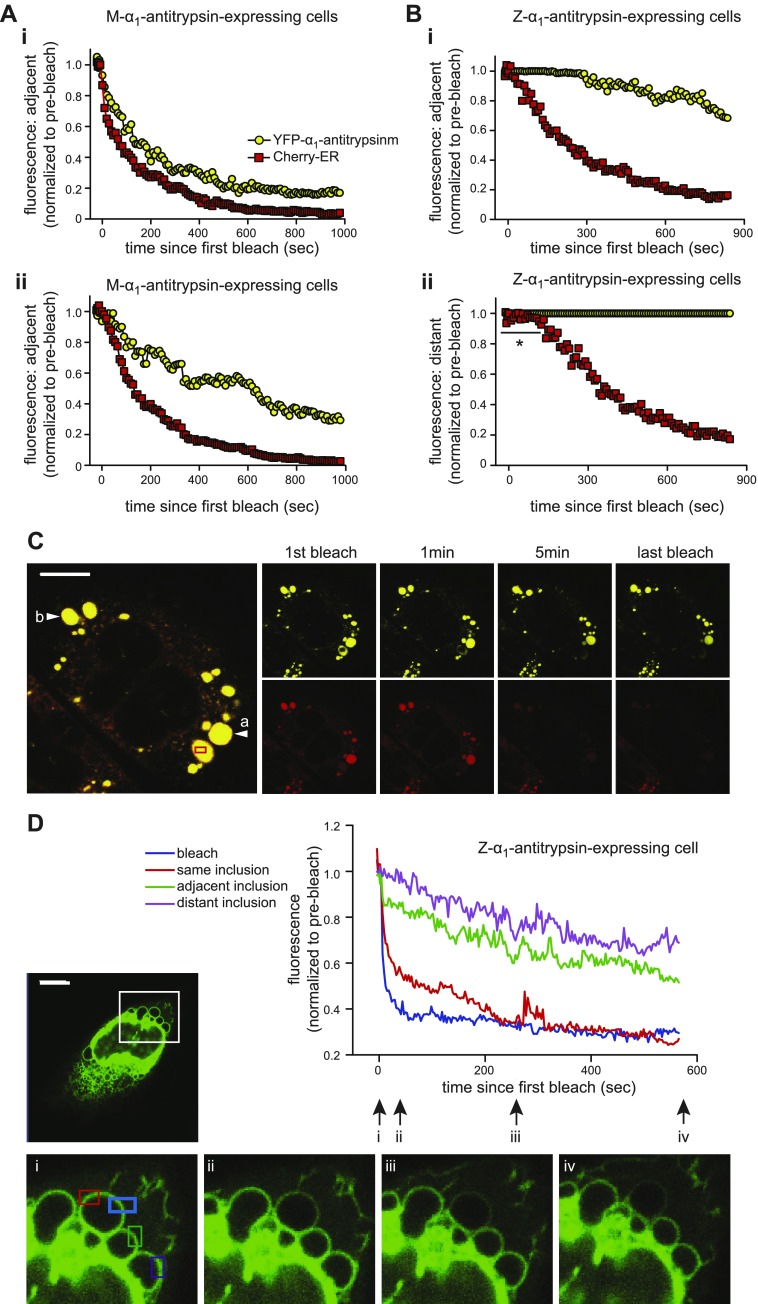
Neither lumens nor membranes of inclusions are connected. *A*) FLIP in CHO cells expressing M-α_1_-antitrypsin (yellow circles) and mCherry-ER (red squares). Single region of interest of 5–10% area of cell was bleached repetitively, and fluorescence of each fluorophore was measured at adjacent (6 µm) (*i*) and distant (40 µm) (*ii*) sites within cell. Both mCherry and YFP fluorescence was immediately lost throughout cell. *B*) FLIP of CHO cells expressing YFP-Z-α_1_-antitrypsin (yellow circles) and mCherry-ER (red squares). Region of interest within single inclusion was repetitively bleached, and fluorescence was measured in adjacent (*i*) and distant (*ii*) inclusion. Delay was present before loss of YFP-Z-α_1_-antitrypsin fluorescence from adjacent inclusion (*i*) *vs*. persistence of fluorescence in distant inclusion (*ii*). Marked delay (indicated with an asterisk) was noted before loss of mCherry-ER fluorescence from distant inclusion (*ii*). *C*) Representative images of YFP-Z-α_1_-antitrypsin photobleaching experiment: bleaching region of interest (red box); adjacent inclusion (arrow *a*), distant inclusion (arrow *b*). Scale bar, 10 µm. *D*) FLIP of cells expressing untagged Z-α_1_-antitrypsin and ER membrane marker cytERM-msfGFP. Graph illustrates cytERM-msfGFP fluorescence at bleached area (blue); 2 equidistant regions of interest, one on same inclusion (red); one on adjacent inclusion (green); and another on distant inclusion (purple). Inset: whole cell and 4 high-powered views of bleaching area at times *i* to *iv* marked on graph. Scale bar, 10 µm.

Because inclusions exchanged contents over a time frame slower than expected by simple diffusion, we wished to investigate the nature of their connectivity. We subjected cells to 2-color FLIP, and as we have shown previously (8), in cells expressing nonpolymerizing YFP-M-α_1_-antitrypsin, the loss of both YFP-M-α_1_-antitrypsin and mCherry-ER fluorescence occurred rapidly from an area adjacent to the bleach (≈6 µm; **Fig. 4*Ai***) but was modestly delayed at a point more distant from the site of bleaching (≈40 µm; Fig. 4*Aii*). The relative mobilities of mCherry-ER and YFP-M-α_1_-antitrypsin were similar over short distances, but YFP-M-α_1_-antitrypsin bleached more slowly at more distant locations. The loss of mCherry-ER fluorescence in cells expressing YFP-M-α_1_-antitrypsin was more rapid than that of YFP-Z-α_1_-antitrypsin even over short distances (Fig. 4*Ai, Bi*). Moreover, in cells expressing YFP-Z-α_1_-antitrypsin, we observed a striking lag period before the loss of mCherry-ER fluorescence from distant inclusions (Fig. 4*Bii,* 4*C*, arrow *b*). This raised the possibility that the continuity of the ER structure might be altered by the accumulation of YFP-Z-α_1_-antitrypsin. To test this, we used cytERM-msfGFP, an ER membrane protein with a short luminal tail such that ER environment should not affect its mobility (21). CHO cells were made to express cytERM-msfGFP and untagged Z-α_1_-antitrypsin; FLIP was then performed. The membrane of one inclusion was repeatedly bleached (Fig. 4*Di*, blue box), and fluorescence was measured in the membrane of the same inclusion (Fig. 4*Di*, red box) and neighboring inclusions (Fig. 4*Di*, green and purple boxes). Marked differences in the loss of fluorescence were observed between the bleached inclusion and its neighbors, even when the neighbor was equidistant from the bleach site (Fig. 4*D*, red and green traces). GFP fluorescence was lost rapidly from the bleached membrane but more slowly from the membrane of adjacent or distant inclusions.

**Figure 5.**
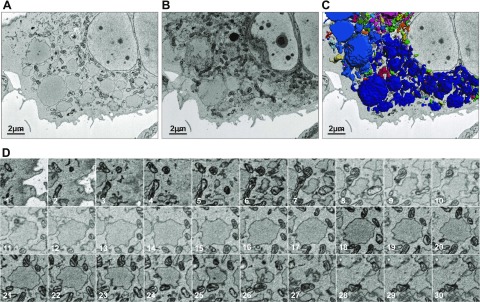
There is no evidence of interinclusion luminal connectivity by electron microscopy. *A*, *B*) Inclusion-laden CHO cell transfected with YFP-Z-α_1_-antitrypsin and selected using fluorescence microscopy was subjected to serial block-face scanning electron microscopy, single scan (*A*), and 3-D reconstruction (*B*). *C*) Peripheries of Z-α_1_-antitrypsin-containing inclusions were imaged using Gatan 3View system and combined using Imaris software to generate 3-D projection by isosurface rendering with surface area detail of 36 nm. *D*) Serial block-face scanning electron microscopy images through inclusion of Z-α_1_-antitrypsin. Lack of interinclusion luminal connectivity is evident.

In an effort to identify physical connections between inclusions and between inclusions and the ER network, we performed 3-dimensional reconstructions of confocal fluorescence images through cells expressing either M- or Z-α_1_-antitrypsin (Supplemental Movies 2 and 3). Although the narrow tubules of peripheral ER were readily seen in cells expressing M-α_1_-antitrypsin, no such connections were observed between inclusions in Z-α_1_-antitrypsin-expressing cells. Next, cells expressing YFP-Z-α_1_-antitrypsin were subjected to serial block-face scanning electron microscopy (**Fig. 5**). The surface of Z-α_1_-antitrypsin inclusions (Fig. 5*A*) were traced in 2-D images and combined to generate a 3-D projection by isosurface rendering with a surface area detail of 36 nm (Fig. 5*B, C* and Supplemental Movie 4). Distinct coloration of physically separated inclusions showed that many of these structures contacted one another (dark blue inclusions in Fig. 5*C* and Supplemental Movie 4). However, inspection of the original 3View stack revealed that inclusion membrane contacts were almost never accompanied by evidence of interluminal connectivity (Fig. 5*D*). The captured resolution of 18 × 18 × 60 nm strongly argues against inclusion connectivity on the scale of conventional tubular ER [diameter of 50–130 nm (22)].

Taken together, these observations suggest that inclusions are physically separated structures that exchange soluble luminal contents despite a lack of obvious luminal connections.

### Inclusion contents can be exchanged despite their physical separation

**Figure 6.**
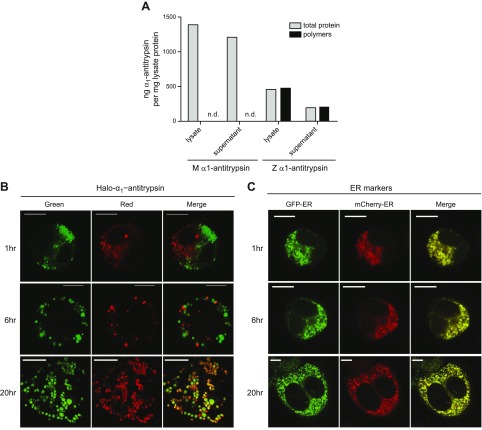
Inclusion contents can be exchanged despite physical separation. *A*) CHO cells were transfected with HaloTag-M-α_1_-antitrypsin or HaloTag-Z-α_1_-antitrypsin. Whole-cell lysates and supernatants were analyzed for total α_1_-antitrypsin and polymer production by sandwich ELISA using polymer-specific 2C1 antibody. Only HaloTag-Z-α_1_-antitrypsin expressing cells are able to produce polymers. *B*) CHO cells were transfected with HaloTag-Z-α_1_-antitrypsin for 24 h, then stained either with TMR (red) or Oregon Green (green). Cells were washed, mixed, and electrofused, then replated and fixed at 1, 6, and 20 h. Colocalization of red- and green-stained HaloTag-Z-α_1_-antitrypsin occurred only after 20 h electrofusion. *C*) CHO cells were cotransfected with untagged Z-α_1_-antitrypsin and either GFP-ER (green) or mCherry-ER (red) for 24 h. Cells were mixed, electrofused, and replated, then fixed at 1, 6, and 20 h. Colocalization of GFP-ER or mCherry-ER-labeled inclusions occurred after only 1 h of electrofusion. Scale bars, 10 µm.

To test whether the contents of physically separated inclusions could mix, we generated HaloTag-M and HaloTag-Z-α_1_-antitrypsin. The HaloTag is a catalytically inactive hydrolase derivative that contains a binding pocket for a set of fluorescent colored ligands, allowing posttranslational covalent labeling of fusion proteins. Radioactive pulse-chase experiments had demonstrated that untagged, YFP-tagged, and Halo-tagged M- and Z-α_1_-antitrypsin each showed similar retention and secretion patterns when expressed in cells (Fig. 1*B, C*). In each case, the M protein was detectable in the medium by 1 h, while Z-α_1_-antitrypsin was detectable in the medium at low levels only after 3 h. When expressed transiently in CHO cells and analyzed by ELISA, HaloTag-M-α_1_-antitrypsin was detectable in cell lysate and extracellular medium but failed to form polymers detectable by the polymer-selective 2C1 antibody (**Fig. 6*A***). By contrast, polymers of HaloTag-Z-α_1_-antitrypsin could be detected both in the cell lysate and medium. We expressed HaloTag-Z-α_1_-antitrypsin in 2 populations of CHO cells, staining one population green with the Oregon Green ligand and the other population red with the TMR ligand. Excess dye was washed away, and cells were recovered by trypsinization before being mixed and subjected to electrofusion using a Gene Pulser II electroporator. At 1 h after electrofusion, cells had reattached to the culture plate sufficiently well to be imaged. At this point, there was minimal spatial mixing of inclusions and no mixing of their luminal contents (Fig. 6*B*). This confirmed that the electrofusion process affected the plasma membrane but did not lead to the fusion of ER membranes acutely. By 6 h, inclusions were more evenly distributed in the cytoplasm, but still there had been little mixing of their contents. However, after 20 h, multiple inclusions were observed to contain both red and green HaloTag-Z-α_1_-antitrypsin, indicating that either vesicular trafficking or fusion of inclusions had occurred. When a similar experiment was performed in cells expressing untagged Z-α_1_-antitrypsin along with the small ER-marker proteins mCherry-ER and GFP-ER, most inclusions already showed evidence of content mixing at the 1 h time point (Fig. 6*C**, top*). Marked differences in fluorophore intensities prevented the examination of the ER markers and the HaloTag constructs within the same cell; nevertheless, these results suggest that in electrofused cells, small ER-resident proteins move swiftly between inclusions, while HaloTag-Z-α_1_-antitrypsin can exchange, but do so far more slowly.

### Transport between inclusions is unrelated to atlastin-mediated homotypic membrane fusion

**Figure 7.**
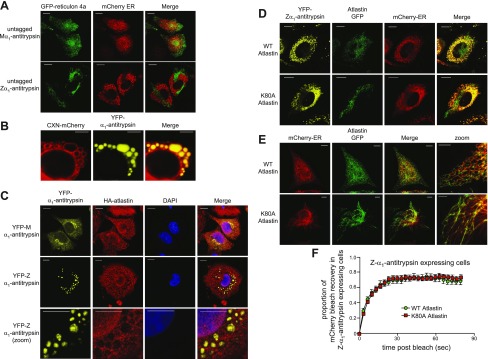
Transport between inclusions is insensitive to dominant negative atlastin. *A*) Lack of colocalization of GFP–reticulon 4a and mCherry-ER: CHO cells were cotransfected with untagged M- or Z-α_1_-antitrypsin, GFP–reticulon 4a (green) and mCherry-ER (red). Reticular pattern is evident of GFP–reticulon 4a and mCherry-ER in M-α_1_-antitrypsin-expressing cells, in contrast to lack of colocalization between tubular GFP–reticulon 4a and punctate mCherry-ER in Z-α_1_-antitrypsin-expressing cells. Scale bars, 10 µm. *B*) Integral membrane protein CNX-mCherry decorates membranes of Z-α_1_-antitrypsin-containing inclusions. Scale bars, 5 µm. *C*) Lack of colocalization of atlastin and YFP-tagged α_1_-antitrypsin: cells were cotransfected with either YFP-M-α_1_-antitrypsin or YFP-Z-α_1_-antitrypsin (yellow) and hemagglutinin (HA)-tagged atlastin, then stained with anti-HA antibody (red). Scale bars, 10 µm. *D*) Lack of colocalization of atlastin-GFP- and YFP-tagged α_1_-antitrypsin: coexpression of GFP-tagged wild-type and K80A mutant atlastin (green), YFP-Z-α_1_-antitrypsin (yellow), and mCherry-ER (red). Poor localization is evident between atlastin-GFP and Z-α_1_-antitrypsin. Scale bars, 10 µm. *E*) Coexpression of GFP-tagged wild-type and K80A mutant atlastin (green) with mCherry-ER (red). Evident is tubular, nonbranching nature of ER in cells expressing mutant atlastin. *F*) mCherry FRAP was performed in CHO cells coexpressing mCherry-ER, Z-α_1_-antitrypsin, and either wild-type or K80A mutant atlastin.

We reasoned that the exchange of luminal contents between inclusions might involve either homotypic ER-ER fusion or recycling *via* ER-derived vesicles. The maintenance of the reticular ER network involves homotypic fusion between ER membranes mediated by homodimerization of atlastin located in each membrane (23, 24). Atlastin is thought to accumulate preferentially at regions of the ER with high membrane curvature in a manner similar to the reticulon scaffolding proteins (25, 26). When we expressed GFP–reticulon 4a in cells expressing untagged M- or Z-α_1_-antitrypsin and the luminal ER marker mCherry-ER, we observed colocalization of GFP–reticulon 4a and the ER marker only in cells expressing M-α_1_-antitrypsin (**Fig. 7*A***, top). In Z-α_1_-antitrypsin-transfected cells, GFP–reticulon 4a was completely excluded from mCherry-ER-containing inclusions, but it did form tubular structures lacking the ER luminal marker (Fig. 7*A*, bottom). Because reticulons (and atlastins) bind only to membranes of high curvature, the failure of reticulon 4a to bind to the flattened surface of Z-α_1_-antitrypsin-positive inclusions suggests a potential mechanism for the fragmentation of the ER into these individual inclusion bodies. Importantly, this contrasted with the localization of an integral ER membrane protein, CNX-mCherry, which, contrary to a previous report (7), decorated the membranes of Z-α_1_-antitrypsin-containing inclusions (Fig. 7*B*).

As we had observed for reticulon 4a, antiatlastin staining failed to label inclusions in Z-α_1_-antitrypsin-expressing cells but rather colocalized with M-α_1_-antitrypsin in a reticular ER pattern (Fig. 7*C*). Moreover, as with GFP–reticulon 4a, colocalization between GFP-tagged atlastin and α_1_-antitrypsin was very poor (Fig. 7*D*). Nevertheless, we tested the effect of expressing the strong dominant negative mutant of atlastin, K80A, on the mobility of mCherry-ER using FRAP. Despite the expected effect of mutant atlastin on ER morphology in control cells (Fig. 7*E*), we observed no difference in mCherry FRAP when wild-type or mutant atlastin were expressed with Z-α_1_-antitrypsin (Fig. 7*F*). Thus, protein exchange between inclusions does not appear to involve atlastin-mediated homotypic ER fusion.

### Communication between inclusions is dependent on cytosol, Sar1, and Sec22B and is sensitive to NEM

**Figure 8.**
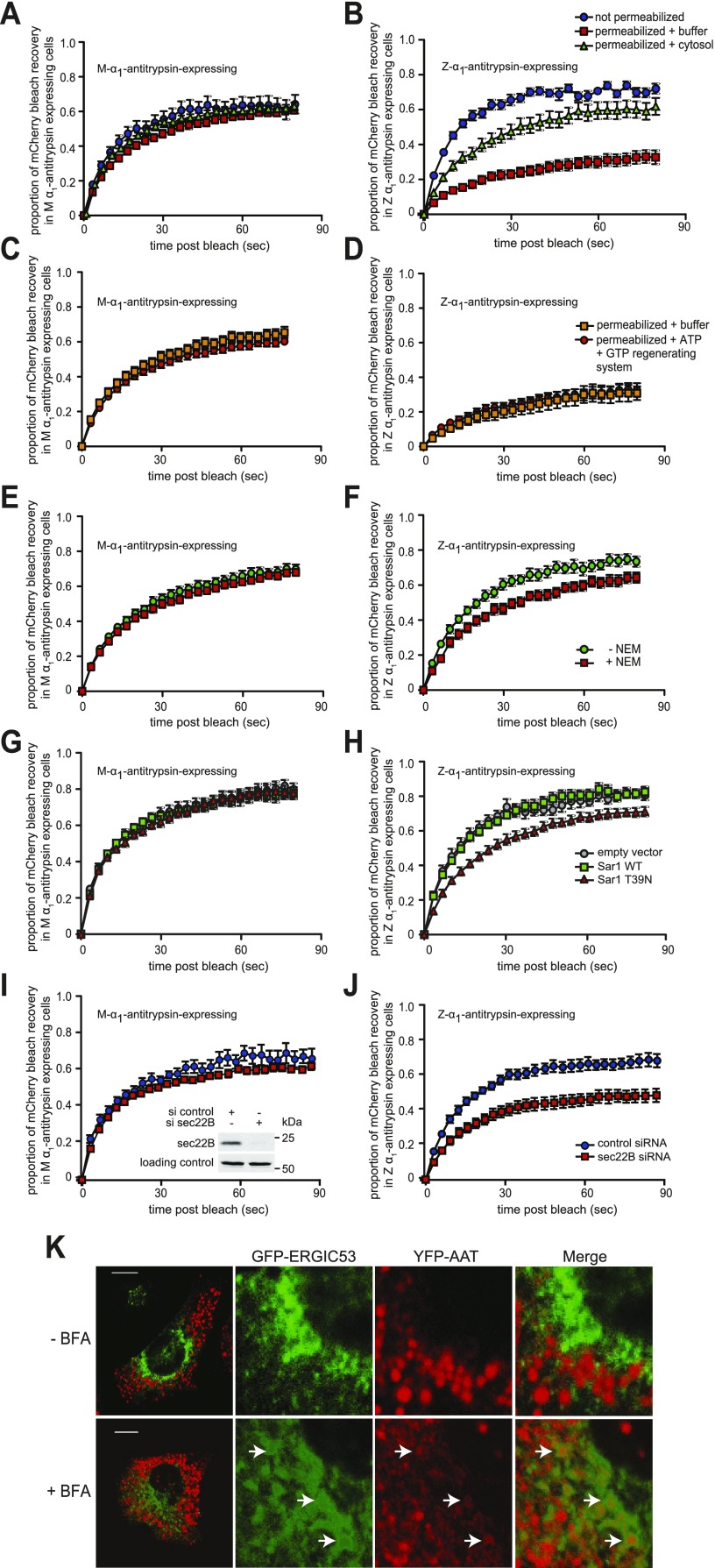
Transport between inclusions is dependent on cytosol, Sar1, and sec22B and is sensitive to NEM. *A*, *B*) mCherry FRAP was performed in CHO cells coexpressing mCherry-ER and either YFP-M-α_1_-antitrypsin (*A*) or YFP-Z-α_1_-antitrypsin (*B*). Cells were placed in ice-cold CTB (control) or were permeabilized with digitonin for 5 min to remove cytosol. Control cells were washed with culture medium (blue squares), while permeabilized cells were either washed with TB (red squares) or reconstituted with purified cytosol (green triangles), then subjected to mCherry FRAP. mCherry FRAP was dramatically reduced in Z-α_1_-antitrypsin-exprssing cells in absence of cytosol (*B,* no cytosol *vs*. cytosol added, *P* < 0.0001). *C*, *D*) Cells coexpressing mCherry-ER and either M-α_1_-antitrypsin (*C*) or Z-α_1_-antitrypsin (*D*) were permeabilized and depleted of cytosol subjected to mCherry FRAP either in TB (orange square*s*) or buffer supplemented with ATP/GTP regenerating system consisting of 1 mM ATP, 10 μM GTP, 10 mM phosphocreatine, and 50 μg/ml creatine kinase in CTB (red circles). *E*, *F*) Digitonin permeabilized cells coexpressing mCherry-ER and either M-α_1_-antitrypsin (*E*) or Z-α_1_-antitrypsin (*F*) were treated with cytosol prepared from cells treated with 20 mM NEM (red squares) or untreated cells (green circles). Cytosol from NEM-treated cells is significantly impaired in supporting mCherry mobility (*F, P* < 0.0001). *G*, *H*) CHO cells cotransfected with mCherry-ER and either M-α_1_-antitrypsin (*G*) or Z-α_1_-antitrypsin (*H*) and either empty vector, wild-type (WT) Sar1 or dominant negative Sar1-T39N were subjected to mCherry FRAP (empty *vs*. WT, *P* = ns; WT *vs*. Sar1-T39N, *P* < 0.0001). *I*, *J*) CHO cells cotransfected with mCherry-ER and either M-α_1_-antitrypsin (*I*) or Z-α_1_-antitrypsin (*J*) and either control siRNA or siRNA targeting Sec22B were subjected to mCherry FRAP (*J,* control *vs*. s22B siRNA, *P* < 0.0001). Inset: immunoblot demonstrates degree of depletion of sec22B. *K*) CHO cells were cotransfected with YFP-Z-α_1_-antitrypsin (red) and GFP-ERGIC-53 (green). Images were acquired using Quasar acquisition and linear unmixing to separate YFP and GFP channels. Cells treated with 10 µg/ml brefeldin A contained population of luminal YFP-Z-α_1_-antitrypsin-positive and membrane GFP-ERGIC-53-positive structures (arrows). Scale bars, 10 µm.

To test whether cytosol was required for interinclusion trafficking, cells were permeabilized with digitonin for 5 min and their cytosol allowed to leak out. In this condition, the recovery of mCherry-ER fluorescence remained almost unchanged in cells expressing M-α_1_-antitrypsin (**Fig. 8*A***). In contrast, the initial rate of recovery of mCherry-ER fluorescence in cells expressing Z-α_1_-antitrypsin fell from 0.066 ± 0.003 to 0.019 ± 0.002 s^−1^, but it was partially rescued by the addition of exogenous cytosol to 0.036 ± 0.004 s^−1^ (Fig. 8*B*). The fractional recovery of fluorescence at 80 s was reduced by cytosol washout from 0.72 ± 0.03 (nonpermeabilized) to 0.33 ± 0.04 (permeabilized with buffer only), but rescued to 0.62 ± 0.05 by exogenous cytosol. To test whether this reflected loss of ATP or GTP, we provided an ATP/GTP regenerating system to the permeabilized cells, but this had no effect on the mobility of mCherry (Fig. 8*C, D*). *N*-Ethylmaleimide-sensitive factor (NSF) is essential to maintain soluble NSF attachment protein receptor (SNARE)-mediated vesicular trafficking (27) but can be inactivated by the thiol-reactive compound NEM (28). We generated cytosol from NEM-treated cells and tested its ability to rescue interinclusion trafficking in the permeabilized cell system. This was significantly impaired at rescuing the trafficking between inclusions in Z-α_1_-antitrypsin-expressing cells but did not affect M-α_1_-antitrypsin cells (Fig. 8*E, F*).

Sar1 is a protein required for the assembly of coat protein complex II (COPII)-coated vesicles and thus protein transport out of the ER (29). Overexpression of the dominant negative GDP-locked Sar1-T39N inhibits vesicle budding from the ER and, as a result of inhibition of forward transport during ER-Golgi recycling, results in redistribution of Golgi-resident proteins to the ER (30, 31). Cells expressing Sar1-CFP T39N were significantly impaired at trafficking mCherry between inclusions compared to cells transfected with empty vector of wild-type Sar1-CFP (Fig. 8*G, H*).

The RNA interference–mediated knockdown of some factors involved in vesicular trafficking from the ER, *e.g.,* syntaxin 5, was too toxic to permit analysis of their involvement in protein mobility between inclusions. However, perhaps owing to some redundancy between the sec22 family members, depleting cells of sec22B using RNA interference was well tolerated (Fig. 8*I*, inset). Sec22B is an ER localized R-SNARE previously implicated in constitutive trafficking from the ER to Golgi apparatus (14, 32). In cells expressing M-α_1_-antitrypsin, loss of sec22B had little effect on the mobility of mCherry-ER, but in cells expressing Z-α_1_-antitrypsin, loss of sec22B caused a selective reduction of mCherry-ER FRAP (Fig. 8*I, J*).

Proteins recycle between the ER and Golgi *via* anterograde COPII-coated vesicles and retrograde COPI vesicles (33). The retrograde trafficking requires the small GTP-binding protein ARF1, which can be inhibited by brefeldin A (34). Pretreatment of cells with 10 μg/ml brefeldin A caused negligible change to the recovery of ER-mCherry fluorescence in cells expressing Z-α_1_-antitrypsin (data not shown). However, it is known that proteins continue to cycle between the ER–Golgi intermediate compartment (ERGIC) and the ER in brefeldin A–treated cells (35). It is therefore of interest that brefeldin A caused small quantities of YFP-Z-α_1_-antitrypsin to accumulate in ERGIC-53-positive structures (Fig. 8*K*). These findings suggest that trafficking between inclusions does not involve recycling *via* the *cis*-Golgi but raise the possibility that trafficking of ER contents between inclusions might involve passage through the ERGIC. Subsequent studies will be required to define this.

Taken together, these experiments indicate that trafficking of soluble protein between inclusions in Z-α_1_-antitrypsin-expressing cells requires cytosolic factors including Sar1 and sec22B and is sensitive to the SNARE inhibitor NEM.

## DISCUSSION

The propensity for PiZZ-homozygous individuals to develop cirrhosis and hepatocellular carcinoma has led to much interest into how the accumulation of intracellular Z-α_1_-antitrypsin perturbs hepatocyte function. Intracellular inclusions containing polymers of Z-α_1_-antitrypsin are ER-like in nature yet appear distinct from the main ER network. In this study, we sought to clarify whether these inclusions are physically or functionally connected to the ER. Despite these structures containing immobilized Z-α_1_-antitrypsin, we observed that small ER-resident proteins are mobile both within and between physically separated inclusions, although the chaperone BiP is less mobile in the presence of Z-α_1_-antitrypsin than in M-α_1_-antitrypsin expressing cells. This is consistent with our hypothesis that impaired chaperone mobility may predispose cells containing polymerized Z-α_1_-antitrypsin to exaggerated ER stress (8).

Despite the absence of a detectable physical connection between some inclusions, we demonstrated mixing of their contents. In the current study, we have been unable to demonstrate luminal connections between inclusions. Although formally such connections might exist, the resolution of our electron microscopy data (18 nm) indicate they would need to be considerably smaller than typical ER tubules of approximately 100 nm diameter. At least for larger inclusions, our current study suggests that some of these structures do not share physically linked membranes. It remains possible that such connections may persist between smaller inclusions, allowing some protein transport *via* diffusion through narrow-necked ER conduits. It was not possible to prove the absence of transient luminal connections between larger inclusions. The requirement for cytosol for efficient movement of ER-mCherry in the permeabilized cell experiments (Fig. 8*B*) argues in favor of vesicular transport, but even without the addition of exogenous cytosol, 45% of mCherry fluorescence recovered after bleaching. This is consistent with a model in which communication between inclusions involves both the vesicular trafficking we detected and luminal connections that we could not observe by electron microscope tomography.

The atlastins are responsible for maintaining the reticular network of the ER through mediating ER-ER homotypic fusion (24). We found no evidence for the involvement of atlastin in the exchange of contents between inclusions. Indeed, we were unable to demonstrate atlastins or reticulons on the surface of inclusions even when overexpressed. These proteins insert into membranes of high curvature *via* their hairpinlike hydrophobic domains, and in so doing, the reticulons maintain the morphology of tubules and sheets, while atlastins promote the formation of reticular ER (24, 26). It is possible that the low angle of curvature of regions of the ER distended by Z-α_1_-antitrypsin polymers is directly responsible for excluding these proteins. This suggests a membrane curvature model for the fragmentation of the ER during the accumulation of Z-α_1_-antitrypsin in which the exclusion of atlastins from portions of the ER prevents the maintenance of ER connectivity. It is noteworthy that despite one report that CNX is excluded from Z-α_1_-antitrypsin containing inclusions (7), in our system, mCherry-tagged CNX was localized to inclusion membranes. It will be valuable to examine endogenous CNX localization in liver tissue from PiZZ individuals to determine which observation reflects true pathophysiology.

Previously we described differences in the rate of protein mobility within the ER of cells expressing Z-α_1_-antitrypsin compared to control cells expressing M-α_1_-antitrypsin (8). The nature of the 2-color photobleaching in this study necessitated a longer bleaching time than that used in our previous work; this accounts at least in part for the apparently disparate results (not shown). The reason for this remains undetermined. It is possible that during the course of the longer bleaching, more of the most mobile mCherry-ER fraction is bleached, thereby leaving the relatively less mobile protein to diffuse into the region of interest during the recovery, thus reducing the differences seen between M- and Z-α_1_-antitrypsin-expressing cells. In addition, in that study, we expressed M- or Z-α_1_-antitrypsin using a Tet-On system, providing tight control over expression levels. In the current study, transient transfection led to some variability in expression levels, which may also account for the failure to resolve differences in mCherry-ER mobility between M- and Z-α_1_-antitrypsin-expressing cells. This underscores the magnitude of differences that were observed between M- and Z-α_1_-antitrypsin-expressing cells that were observed despite this technical limitation—for example, the dependence on cytosol in permeabilized cell experiments. But an important limitation of our study is the use of an overexpression cell culture system. This is likely to have exaggerated and accelerated ER distension, leading to inclusion body formation. However, inclusion bodies in the hepatocytes of affected PiZZ individuals have similar physical characteristics to those we have studied (36), and so our observations may reflect the behavior of inclusions *in vivo.* Nevertheless, the relevance of our findings to human disease are likely to be restricted to those cells in which inclusion formation is sufficiently advanced to cause fragmentation of the ER.

We observed a requirement for cytosolic component or components in the transport of material between inclusions, as well as sensitivity to NEM, knockdown of sec22B, and the presence of dominant negative Sar1, making a SNARE-mediated transport event involving COPII vesicular transport likely. The appearance of Z-α_1_-antitrypsin in ERGIC-53-positive structures after treatment with brefeldin A suggests that recycling *via* the ERGIC may be involved in the exchange of material between inclusions. In contrast to ER-Golgi recycling, which is blocked by brefeldin A, it has been shown that recycling between the ER and ERGIC persists in the presence of brefeldin A (35). Such a mechanism appears more plausible than direct SNARE-mediated homotypic ER trafficking or fusion, which to date has only been observed in yeast and not in metazoans (37). Subsequent studies will be required to elucidate this mechanism further.

It is feasible that interinclusion ER chaperone exchange may act to minimize ER heterogeneity, thereby protecting inclusion-containing cells from ER stress. Further studies are required to test this directly, and also to establish whether the phenomenon described herein is generalizable to other circumstances of ER fragmentation—for example, in Russell body–expressing plasma cells seen in chronic inflammation and multiple myeloma (38, 39), other serpinopathies such as FENIB (familial encephalopathy with neuroserpin inclusion bodies) (40), and in response to ischemia (41).

Taken together, our findings shed light on the cellular mechanisms by which inclusions formed by the fragmentation of the ER during accumulation of polymerized Z-α_1_-antitrypsin remain able to exchange luminal contents.

## Abbreviations

CHO: Chinese hamster ovary
CNX: calnexin
COPII: coat protein complex II
CTB: complete transport buffer
ER: endoplasmic reticulum
ERGIC: ER–Golgi intermediate compartment
FLIP: fluorescence loss in photobleaching
FRAP: fluorescence recovery after photobleaching
GFP: green fluorescent protein
NEM: *N*-ethylmaleimide
NSF: *N*-ethylmaleimide-sensitive factor
siRNA: short interfering RNA
SNARE: soluble NSF attachment protein receptor
TB: transport buffer
YFP: yellow fluorescent protein
